# Gender differences in the prevalence and correlates of COVID-19 fear among mental health professionals: a network perspective based on a national survey in China

**DOI:** 10.3389/fpsyt.2025.1631050

**Published:** 2025-08-21

**Authors:** Shu-Ying Rao, Mu-Rui Zheng, Feng-Rong An, Yuan Feng, Zhaohui Su, Teris Cheung, Gabor S. Ungvari, Chee H. Ng, Yu-Tao Xiang, Gang Wang

**Affiliations:** ^1^ Unit of Psychiatry, Department of Public Health and Medicinal Administration, & Institute of Translational Medicine, Faculty of Health Sciences, University of Macau, Macao, Macao SAR, China; ^2^ Centre for Cognitive and Brain Sciences, University of Macau, Macao, Macao SAR, China; ^3^ Beijing Key Laboratory of Mental Disorders, National Clinical Research Center for Mental Disorders & National Center for Mental Disorders, Beijing Anding Hospital, Capital Medical University, Beijing, China; ^4^ School of Public Health, Southeast University, Nanjing, China; ^5^ School of Nursing, Hong Kong Polytechnic University, Hong Kong, Hong Kong SAR, China; ^6^ Section of Psychiatry, University of Notre Dame Australia, Fremantle, WA, Australia; ^7^ Division of Psychiatry, School of Medicine, University of Western Australia, Perth, WA, Australia; ^8^ Department of Psychiatry, The Melbourne Clinic and St Vincent’s Hospital, University of Melbourne, Richmond, VIC, Australia

**Keywords:** mental health professionals, gender difference, fear of COVID-19, network analysis, mental health

## Abstract

**Background:**

Gender differences in COVID-19-related fear among mental health professionals (MHPs) have been inadequately studied. This study compared the gender differences in prevalence, correlates and network structure of COVID-19 fear among MHPs in China in the post-pandemic era.

**Methods:**

A nationwide cross-sectional study was conducted between January 22 and February 10, 2023. Binary logistic regression was used to identify factors associated with COVID-19 fear. Expected Influence was used to identify the most central (influential) symptoms in gender-specific networks, while network comparison tests were conducted to assess the differences between male and female models.

**Results:**

Among 7,199 MHPs, the overall prevalence of COVID-19 fear was 63.5% (95% CI: 62.3%-64.6%), with 61.7% (95% CI: 58.9%-64.4%) in males and 65.0% (95% CI: 63.7%-66.2%) in females. For male MHPs, factors associated with COVID-19 fear included having married status (OR: 1.39; 95% CI: 1.02–1.90; P = 0.037), poorer economic status (poor vs. good: OR: 1.91; 95% CI: 1.23–2.98; P = 0.004), more severe insomnia (OR: 1.04; 95% CI: 1.01–1.07; P = 0.018), and depression (OR: 1.09; 95% CI: 1.05–1.12; P < 0.001). For female MHPs, the significant factors included having married status (OR: 1.21; 95% CI: 1.06–1.37; P = 0.004), poorer economic status (poor vs. good: OR: 1.39; 95% CI: 1.11–1.73; P = 0.004), more severe insomnia (OR: 1.05; 95% CI: 1.03–1.06; P < 0.001), depression (OR: 1.09; 95% CI: 1.08–1.11; P < 0.001), and quarantine experience (OR: 1.17; 95% CI: 1.04–1.30; P = 0.006). Network analysis revealed that the most central symptom in the male network was FOC6 (sleep difficulties due to COVID-19 concerns), while the corresponding node in the female network model was FOC7 (palpitations when thinking about COVID-19).

**Conclusion:**

COVID-19-related fear was more prevalent among female MHPs than males. Specific interventions targeting the central symptoms in each network should be implemented to alleviate COVID-19 fear effectively and improve the mental health of MHPs in the post-pandemic era.

## 1 Introduction

In China, approximately 50% of the population had been infected COVID-19 by 2023 (1). The clinical impact of COVID-19 extends beyond the acute viral infection phase itself. Apart from physical symptoms, COVID-19 often leads to psychological problems and evokes widespread fear among the population. Fear associated with COVID-19 manifests across four dimensions: physical, interpersonal, cognitive and behavioral (2). The development of post-infection sequelae of COVID-19 (PISC), commonly referred to as Long COVID, is an emerging public health concern. Certain symptoms, such as sleep disturbances, myalgia, anosmia, dysgeusia, and headaches, often persist beyond several months, adversely impacting one’s long-term mental health and quality of life (3, 4). Therefore, it is crucial to examine the long-term influence of the pandemic on mental health among the population in the post-COVID era such as fear of COVID-19.

The mental health effects of the pandemic among mental health professionals (MHPs) have been previously studied (5–7). A study conducted in the Netherlands found that 30% of MHPs in mental health institutions exhibited symptoms of depression, while 4.2% had considered leaving their positions during the pandemic (8). In China, MHPs predominantly comprise psychiatrists, nurses, or technicians, working in psychiatric hospitals or psychiatric departments of general hospitals (9). These professionals provide most of the mental health services in China (10). Notably, their clinical practice often occurs without specialized respiratory protection, a critical measure for COVID-19 prevention. Such resource limitation may exacerbate anxiety among MHPs when encountering potential COVID-19 cases. Previous studies on MHPs during the pandemic mostly focused on symptoms such as insomnia, anxiety and depression, and few of them addressed the prevalence and correlates of COVID-19-related fear, particularly in the post-COVID era. However, COVID-19 fear might indicate underlying mental health problems, for instance, a previous meta-analysis revealed that COVID-19 fear was significantly related with anxiety and depression, especially among healthcare professionals (HPs) (11).

Apart from the COVID-19 infection itself, the implementation of strict public health preventive measures during the pandemic might have prolonged adverse socio-occupational impacts on MHPs even in the post-pandemic era. These effects could have both psychological and physiological manifestations, such as depressive and anxiety symptoms, sleep disturbances, palpitations, and post-traumatic stress symptoms, with many HPs reporting persistent fear when recalling their experiences during the pandemic (12). Furthermore, the heightened prevalence of mental health problems among the population during and immediately after the pandemic would significantly increase the workload of MHPs, thereby exacerbating the risk of mental health problems including COVID-19 fear. Moreover, compared to most of other HPs, MHPs were exposed to patients’ pandemic-related trauma narratives even during the post-pandemic period, potentially leading to vicarious fear (13). Such sustained exposure might result in empathic overload that could affect their psychological well-being. Among the professional development skills for MHPs, the physiological consequences of emotion regulation might have unwanted impacts. Previous research indicated that emotion suppression strategies, often employed in clinical settings, might elevate diurnal cortisol secretion, a biomarker of chronic stress (14). Striving to suppress emotions could increase cortisol concentration, which might paradoxically amplify pandemic-related distress (15). Thus, understanding COVID-19-related fear among MHPs is crucial for addressing their mental well-being, professional competence, work satisfaction and retention, which might serve as a model for other HPs and ultimately benefiting patient care.

Previous research found gender differences in the prevalence and experience of long COVID, with females being more likely to report persistent symptoms compared to males (16). This might be attributed to females being more prone to experience symptoms of mood and hyper-arousal (17). However, previous findings have been inconsistent. Some studies reported that females had higher levels of COVID-19-related fear than males (18, 19). Conversely, a study found that male HP expressed greater fear of COVID-19 than their female counterparts (20), while another study reported no significant gender differences in HPs during the pandemic (21). Such inconsistencies suggest that gender differences in COVID-19-related fear might vary across different populations and change over time. Therefore, it is important to further clarify gender differences in COVID-19-related fear.

It should be noted that most studies evaluated COVID-19 fear based solely on total scores of standard scales, weighing individual symptoms equally (22, 23). However, this approach obscures the meaningful inter-relationships between individual symptoms and hinders the identification of influential symptoms that might play a more significant role in the maintenance of COVID-19 fear. To address this limitation, network analysis has been used as a novel statistical method that could examine how psychiatric symptoms interact with each other and identify the most central (influential) symptoms (24). Network models consist of nodes and edges; nodes represent psychiatric symptoms, while edges reflect their statistical associations (25). Using such an approach, it is possible to identify the most central nodes whose activation could directly influence nearby connected nodes (26), potentially preventing the exacerbation of certain disorders through targeted attention to these nodes (27). Network analysis concerning the COVID-19 fear have been applied to the study of the general population (28), as well as forensic healthcare workers (28).

There are several common limitations among previous studies. First, most have primarily focused on mental health issues, such as anxiety and depression, among MHPs, with limited attention given to COVID-19-related fear, particularly in the post-pandemic context. Second, gender differences in COVID-19-related fear remain poorly understood, with available findings showing inconsistencies across different populations. Third, previous research on COVID-19-related fear has predominantly relied on aggregate scale scores, neglecting symptom-level interactions, and thus failing to identify the most influential symptoms. To address these gaps, we compared gender differences in the prevalence, correlates and network structure of COVID-19-related fear among MHPs in the post-pandemic era.

## 2 Methods

### 2.1 Study design and participants

The study assessed the mental health status of MHPs who experienced COVID-19 fear in the post-pandemic era. We conducted a cross-sectional national survey from January 22 to February 10, 2023, utilizing a snowball sampling method. A total of 7,199 MHPs across China participated in the survey. Following previous studies (29, 30), we employed the “Questionnaire Star” program, which is widely used in epidemiological research in public mental health in China (31–33). The program generated a Quick Response QR) Code for a specific questionnaire, enabling participants to scan and share the survey via WeChat, one of the most widely used social messaging platforms in China. Furthermore, WeChat served as a unique tool for reporting daily health status during the pandemic in China (34), ensuring widespread use among MHPs nationally.

Eligible participants were: (1) Individuals aged 18 years or older; (2) MHPs, including psychiatrists, nurses, or technicians, working in psychiatric hospitals or psychiatric departments of general hospitals across China; (3) Individuals who had recovered from COVID-19 by December 1, 2022; (4) MHPs with post-infection sequelae (PISC) of COVID-19; and (5) Individuals who could comprehend Chinese and provide electronic written informed consent. Exclusion criteria included: (1) Individuals with severe pre-existing health conditions (e.g., severe cardiovascular disease, respiratory disorders or immunosuppression) prior to the pandemic; (2) Pregnant individuals; and (3) MHPs who were not on duty during the COVID-19 pandemic. The Ethics Committee of Beijing Anding Hospital approved the study protocol (“The Linshenwei No.: 26 in 2023”), and all participants provided electronic informed consent.

### 2.2 Measures and assessments

The sociodemographic and health-related information of MHPs were collected, including age, gender, marital status, working years, living status, economic and health status, COVID-19 infection history, and quarantine experience. Fear-related symptoms of MHPs were evaluated with the validated Chinese version of the Fear of COVID-19 Scale (FCV-19S) (35, 36), which consists seven items: (1) FOC1: Afraid of COVID-19 pandemic; (2) FOC2: Uncomfortable to think about COVID-19 pandemic; (3) FOC3: Clammy when thinking about COVID-19 pandemic; (4) FOC4: Afraid of losing life because of COVID-19 pandemic; (5) FOC5: Nervous when watching news about COVID-19 pandemic; (6) FOC6: Sleep difficulties caused by worried about COVID-19; (7) FOC7: Palpitation when thinking about COVID-19 pandemic. The FCV-19S items are categorized into two domains: physical response (FOC3, FOC6 and FOC7) and fear-related thoughts (FOC1, FOC2, FOC4 and FOC5). All seven items are rated on a 5-point Likert scale, with total scores ranging from 7 to 35, where higher scores indicate greater dread (36). Following the previous studies, a cutoff score of ≥ 16 was used to define as having severe fear of COVID-19 pandemic (37, 38).

Depressive symptoms were assessed with the validated Chinese version of the nine-item Patient Health Questionnaire (PHQ-9) (39, 40). The PHQ-9 measures specific depression symptoms such as poor mood, loss of interest, and sleep disturbances (41). Each item is rated from 0 (not at all) to 3 (nearly every day), with a total score ranging from 0 to 27. A higher total score indicates a greater degree of depression.

Anxiety symptoms were measured using the validated Chinese version of the General Anxiety Disorder -7 (GAD-7) (42, 43). The GAD-7 consists of seven items, and each item is scored from 0 (not at all) to 3 (nearly every day) with a total score ranging from 0 to 21. The items correspond to specific anxiety symptoms, including nervousness, anxiety, and excessive worry.

Insomnia symptoms were evaluated using the validated Chinese version of the Insomnia Severity Index (ISI-7) (44, 45), which comprises seven items, and each is rated from 0 (no problem) to 4 (very severe problem). The total score ranges from 0 to 28, with higher scores indicating more severe insomnia symptoms. Each ISI-7 item assesses specific insomnia symptoms, such as difficulty falling asleep, maintaining sleep, and early awakenings.

These scales had satisfactory psychometric properties in studies among Chinese populations. In this study, the Cronbach’s alpha values for the FCV-19S, PHQ-9, GAD-7 and ISI-7 were 0.93, 0.90, 0.93, 0.94, respectively.

### 2.3 Statistical analysis

All statistical analyses were performed using the R software program (Version 4.4.2) (46). The normality of distributions was assessed using Q-Q plot through the “ggpubr” package (Version 0.6.0) (47). Continuous variables that followed a normal distribution were compared between the groups with and without COVID-19 fear using t-test, while non-normally distributed variables were compared using Mann Whitney U test. Chi-square tests were employed for categorical variables. Additionally, a binary logistic regression was conducted to identify the factors that were significantly associated with COVID-19 fear in MHPs with statistically significant variables from the univariate analyses serving as independent variables. A two-tailed significance level of P < 0.05 was used for all tests in this study.

In the network model, each node represented a specific COVID-19 fear-related symptom, with edges reflecting the associations between neighboring nodes. Thicker edges indicated stronger relationships between nodes. Green and red color edges represented positive and negative associations, respectively (48). We employed a Graphical Gaussian Model (GGM) to assess partial correlations among COVID-19 fear symptoms, establishing an undirected network model, thus enabling the exploration of nodes independently of causal relationships (49). To produce a sparse model, a graphic Least Absolute Shrinkage and Selection Operator (GLASSO) was applied with a tuning parameter of 0.5 to eliminate spurious edges by setting weak partial correlation coefficients to zero (50). Additionally, we used the Extended Bayesian Information Criterion (EBIC) to select the best-fitting network model (51). Following the EBICglasso procedure, the “EstimateNetwork” function from the “bootnet” package (Version 1.5.6) was used to identify the best-fit network model (49), while “qgraph” (Version 2.1.0) (48) and “ggplot2” (Version 3.5.0) (52) were used for network visualization.

To quantify the most important symptoms in the network, centrality indices were used. Specifically, we utilized the Expected Influence (EI), which rigorously evaluates the cumulative impact of the node’s connections, accounting for both positive and negative edges (27) we used the package “mgm” (Version 1.2.0) (53) to compute the predictability, which indicates the internal connections between a node and its adjacent nodes.

The stability of the established network model was evaluated using the R-package “bootnet”, in which reliability of the weights of each edge in the network was assessed through the bootstrapped 95% confidence interval (95% CI). A narrow bootstrapped 95% CI that does not contain zero suggests a robust network (54). Further, centrality stability was assessed through case-dropping bootstrap and computing the correlation stability coefficient (CS-coefficient). A CS-coefficient above 0.25 indicates a stable result and a value exceeding 0.5 is preferred (49). Moreover, the significance of differences in edge weights and centrality indices was assessed through bootstrapped difference tests. More black boxes suggest that the strength of this connection remains consistent across multiple resampling, therefore the network structure is relatively stable (55).

To investigate potential differences in network characteristics between males and females, we conducted a network comparison test (NCT) using the package “NetworkComparisonTest” (Version 2.2.2) (56). The NCT method compares network structures based on three key components: network structure, edge strength and global strength. Specifically, network structure is assessed by comparing the distribution of edge weights, edge strength examines whether specific edges differ significantly between networks, and global strength represents the sum of all edge weights, thus providing insight into the overall connectivity of the networks (57).

## 3 Results

### 3.1 Participant characteristics

From a total of 11,524 MHPs invited to participate in this study, 7,199 who met the study entry criteria were included for analyses. Demographic characteristics of the participants are summarized in **Table 1**. Participants’ ages ranged from 18 to 66 years, with an average age of 35.1 years (SD = 8.3 years). Notably, over half had experienced at least a week of quarantine during the pandemic (n = 4,117; 57.2%).

**Table 1:** Demographic characteristics of mental health professionals with and without COVID-19 fear grouped by gender.

Variable	Total (N=7,199)		Male				Univariate analyses		Female				Univariate analyses	
			No-fear (N=442)		Fear (N=715)				No-fear (N=2,109)		Fear (N=3,933)			
	N	%	N	%	N	%	*χ* ^2^	P	N	%	N	%	*χ* ^2^	P
Married	5,302	73.6	256	57.9	478	66.9	9.4	**0.002**	1,554	73.7	3,014	76.6	6.4	**<0.001**
Living with others	6,464	89.8	374	84.6	604	84.5	0.004	0.949	1,908	90.5	3,578	91.0	0.4	0.518
At least 1-week quarantine during the COVID-19	4,116	57.2	263	59.5	432	60.7	0.09	0.757	1,114	52.8	2,307	58.7	19.0	**<0.001**
Economic status														
Poor	844	11.7	70	15.8	190	26.6	25.2	**<0.001**	149	7.1	435	11.1	40.1	**<0.001**
Fair	5,963	82.8	334	75.6	496	69.4			1,811	85.9	3,322	84.5		
Good	392	5.4	38	8.6	29	4.1			149	7.1	176	4.5		
Health status														
Poor	586	8.1	34	7.7	106	14.8	25.2	**<0.001**	104	4.9	342	8.7	54.9	**<0.001**
Fair	5,422	75.3	297	67.2	499	69.8			1,588	73.7	3,038	77.2		
Good	1,191	16.5	111	25.1	110	15.4			417	19.8	553	14.1		

	M	SD	M	SD	M	SD	Z	P	M	SD	M	SD	Z	P
Age (years)	35.1	8.3	32.3	8.3	33.7	8.4	-2.8	**0.004**	35.3	8.1	35.6	8.2	-1.6	0.109
Working years	13.0	9.1	9.6	9.0	11.0	8.7	-3.6	**<0.001**	13.4	9.1	13.6	9.0	-0.9	0.322
GAD-7 total	3.6	4.4	2.3	3.6	5.1	5.1	-8.2	**<0.001**	1.9	3.1	4.3	4.6	-8.2	**<0.001**
ISI-7 total	7.1	5.5	5.5	5.2	8.9	6.5	-8.2	**<0.001**	5.2	4.7	7.9	5.5	-8.2	**<0.001**
PHQ-9 total	6.3	5.5	4.7	4.9	8.3	6.3	-8.2	**<0.001**	4.3	4.3	7.2	5.6	-8.2	**<0.001**

	No-fear	Fear	*χ* ^2^	P										
Male	442	715	4.6	0.03										
Female	2,109	3,933												

Bolded values: <0.05; M: mean; SD: standard deviation; GAD-7: the 7-item General Anxiety Disorder; ISI-7: the 7-item Insomnia Severity Index; PHQ-9: the 9-item Patient Health Questionnaire.

P-values are not adjusted for multiple comparisons.

### 3.2 Gender differences in the prevalence of COVID-19 fear in mental health professionals

The overall prevalence of COVID-19 fear (defined as a FCV-19S total score ≥ 16) among MHPs was 63.5% (n = 4,648; 95% CI: 62.3%-64.6%) Specifically, 61.7% (n=715, 95% CI: 58.9%-64.4%) of male participants and 65.0% (n=3,933, 95% CI: 63.7%-66.2%) of female participants reported fear of COVID-19. The gender difference of prevalence of COVID-19 fear reached a significance level (χ^2^ = 4.6; P = 0.03).

### 3.3 Independent correlates of COVID-19 fear among male and female MHPs

**Table 1** shows the demographic characteristics of MHPs with and those without COVID-19 fear by gender.

For males, compared with those without significant COVID-19 fear, MHPs with fear were more likely to be older (P = 0.004), working longer years (P < 0.001), be married (P = 0.002) and reporting poorer economic (P < 0.001) and health status (P < 0.001). Additionally, they exhibited significantly higher mean scores on the PHQ-9 (P < 0.001), GAD-7 (P < 0.001) and ISI-7 (P < 0.001) scales. In contrast, for females, compared with those without COVID-19 fear, MHPs with fear were more likely to be married (P < 0.001), quarantined previously (P < 0.001), and reporting worse economic (P < 0.001) and health conditions (P < 0.001). Similar to males, females with fear scored significantly higher on the PHQ-9 (P < 0.001), GAD-7 (P < 0.001), and ISI-7 (P < 0.001) scales.

Binary logistic regression analysis revealed that, among male MHPs, being married was significantly associated with higher risk of COVID-19 fear (OR: 1.39; 95%CI: 1.02-1.90; P=0.037). Furthermore, having worse economic status (poor vs. good: OR: 1.91; 95%CI: 1.23-2.98; P=0.004) and more severe insomnia (OR:1.04; 95%CI: 1.01-1.07; P=0.018) and depression (OR:1.09; 95%CI: 1.05-1.12; P<0.001) were significantly associated with more severe COVID-19 fear (**Table 2**). Similarly, among female MHPs, having married status (OR: 1.21; 95% CI: 1.06-1.37; P = 0.004), worse economic status (poor vs. good: OR: 1.39; 95% CI: 1.11-1.73; P=0.004) and more severe insomnia (OR: 1.05; 95%CI: 1.03-1.06; P<0.001) and depression (OR: 1.09; 95%CI: 1.08-1.11; P<0.001) were significantly associated with more severe COVID-19 fear. In female but not in male MHPs, quarantine experience (OR: 1.17; 95%CI: 1.04-1.30; P=0.006) was significantly associated with a higher level of COVID-19 fear (**Table 2**). GAD-7 was excluded in the multivariate analyses due to the multicollinearity between PHQ-9 and GAD-7 assessments.

**Table 2:** Independent correlates of COVID-19 fear among male and female mental health professionals.

Variables	Fear of COVID-19(Y/N)					
	Male			Female		
	P	OR	95%CI	P	OR	95%CI
Married	**0.037**	1.39	1.02-1.90	**0.004**	1.21	1.06-1.37
Age	0.400	1.02	0.98-1.05	–	–	–
Economic status	–	–	–	–	–	–
Poor	**0.004**	1.91	1.23-2.98	**0.004**	1.39	1.11-1.73
Fair	>0.900	0.99	0.75-1.30	0.700	0.97	0.85-1.11
Good	–	1.00	–	–	1.00	–
Health status						
Poor	0.600	0.89	0.60-1.32	0.062	0.82	0.67-1.01
Fair	0.700	0.95	0.75-1.21	0.400	0.95	0.84-1.08
Good	–	1.00	–	–	1.00	–
Working years	0.600	1.02	0.96-1.02	–	–	–
At least 1-week quarantine during the COVID-19	–	–	–	**0.006**	1.17	1.04-1.30
ISI-7 total	**0.018**	1.04	1.01-1.07	**<0.001**	1.05	1.03-1.06
PHQ-9 total	**<0.001**	1.09	1.05-1.12	**<0.001**	1.09	1.08-1.11

Bolded value: <0.05; CI, confidential interval; OR, odds ratio.

P-values are unadjusted for multiple comparisons.

### 3.4 Network structure of COVID-19 fear

**Figure 1:**
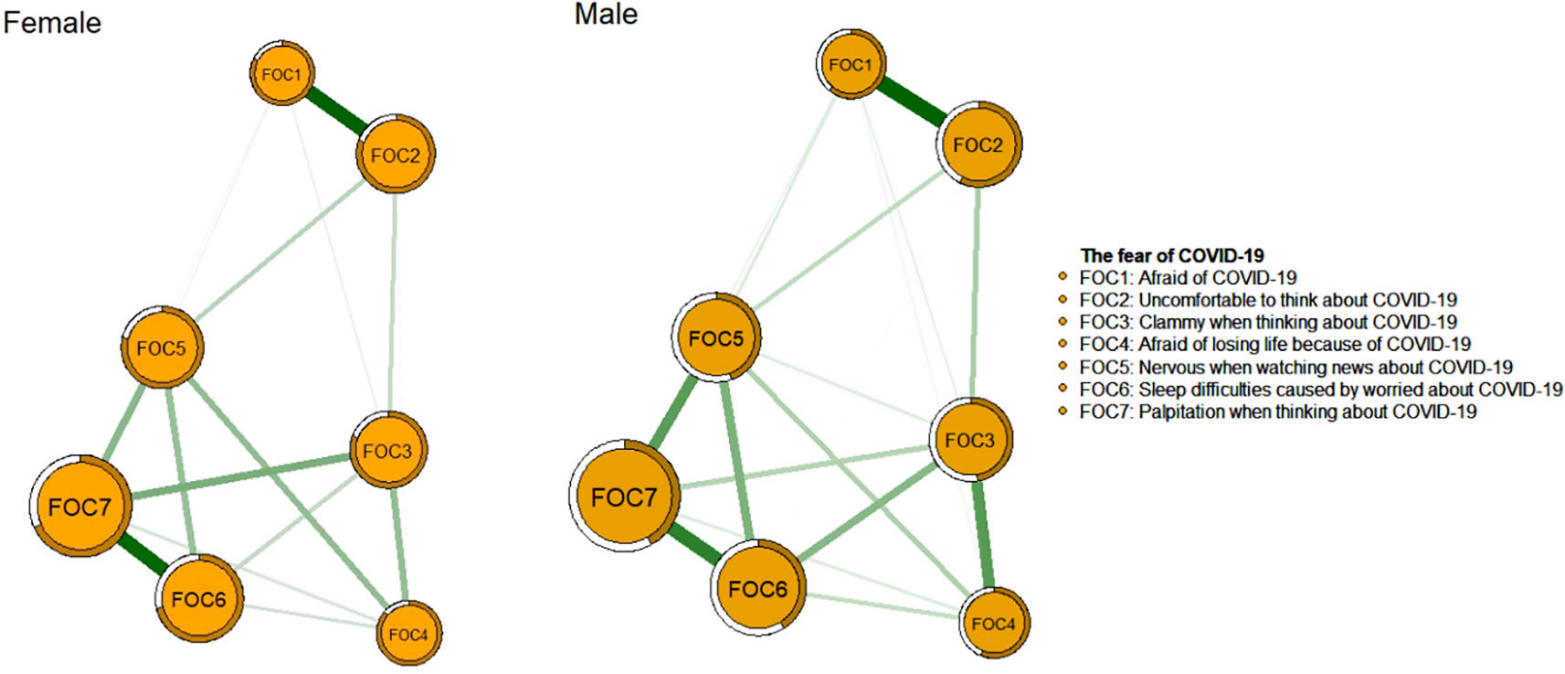
Network structure of COVID-19 fear among female and male mental health professionals.

**Figure 2:**
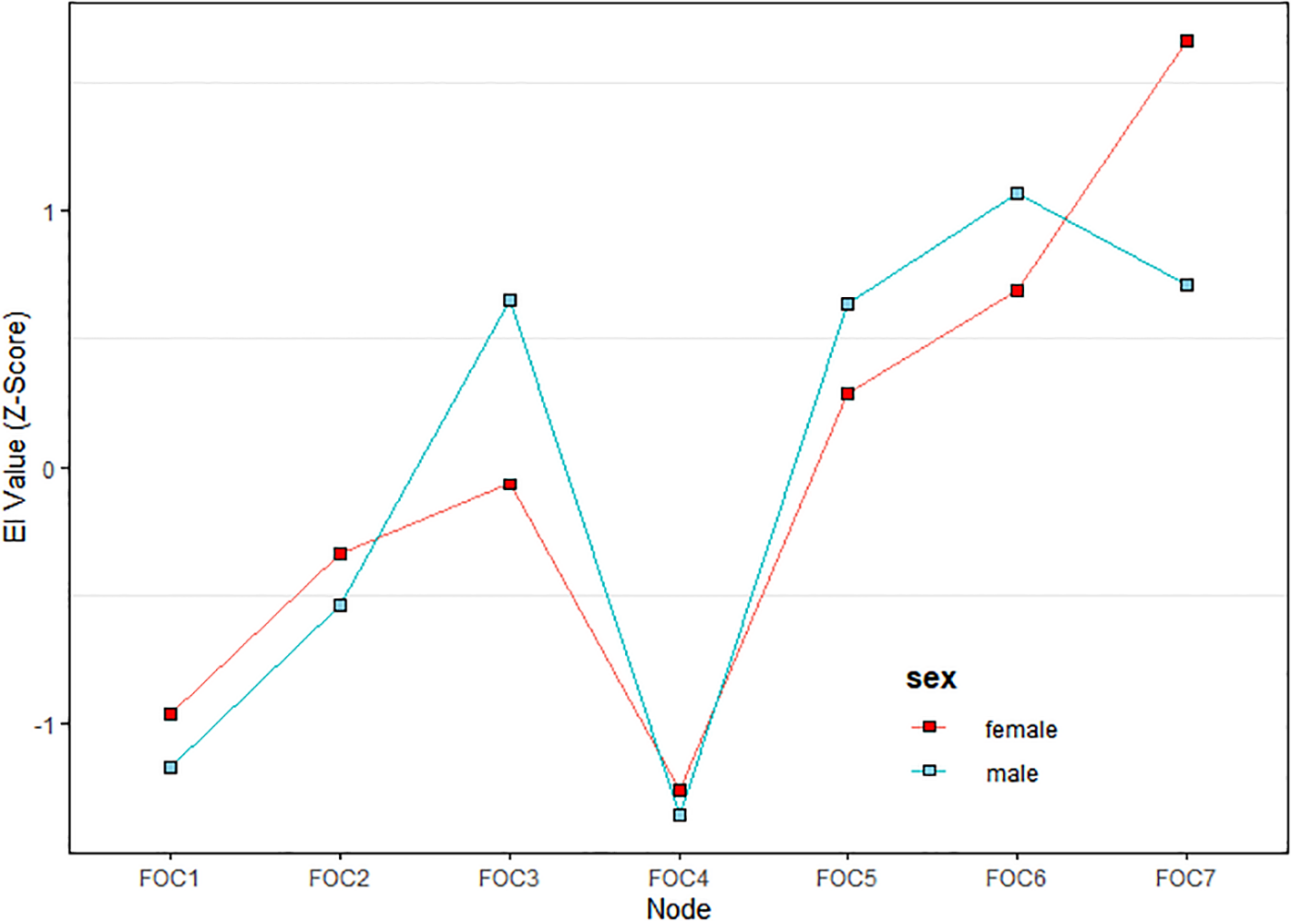
The fear of COVID-19 centrality changes among female and male mental health professionals.

**Figures 1** and **2** illustrate the network structure and centrality indices of COVID-19 fear in male and female MHPs, respectively. In the male model, FOC6 (Sleep difficulties caused by worried about COVID-19 pandemic) had the highest EI value, whereas in the female network, FOC7 (Palpitation when thinking about COVID-19 pandemic) exhibited the highest centrality, indicating that these symptoms were the most influential in the respective models. Predictability is encoded in the outer ring’s filled-to-blank area ratio, where the colored proportion reflects the magnitude of the EI value in **Figure 1**. The mean predictability of the seven nodes was 0.45 for males and 0.74 for female, indicating that an average of 45% and 74% of the variance in each node could be accounted for by its neighboring nodes in the respective models (**Supplementary Table S1**).

### 3.5 Network comparison tests by gender

**Figure 3:**
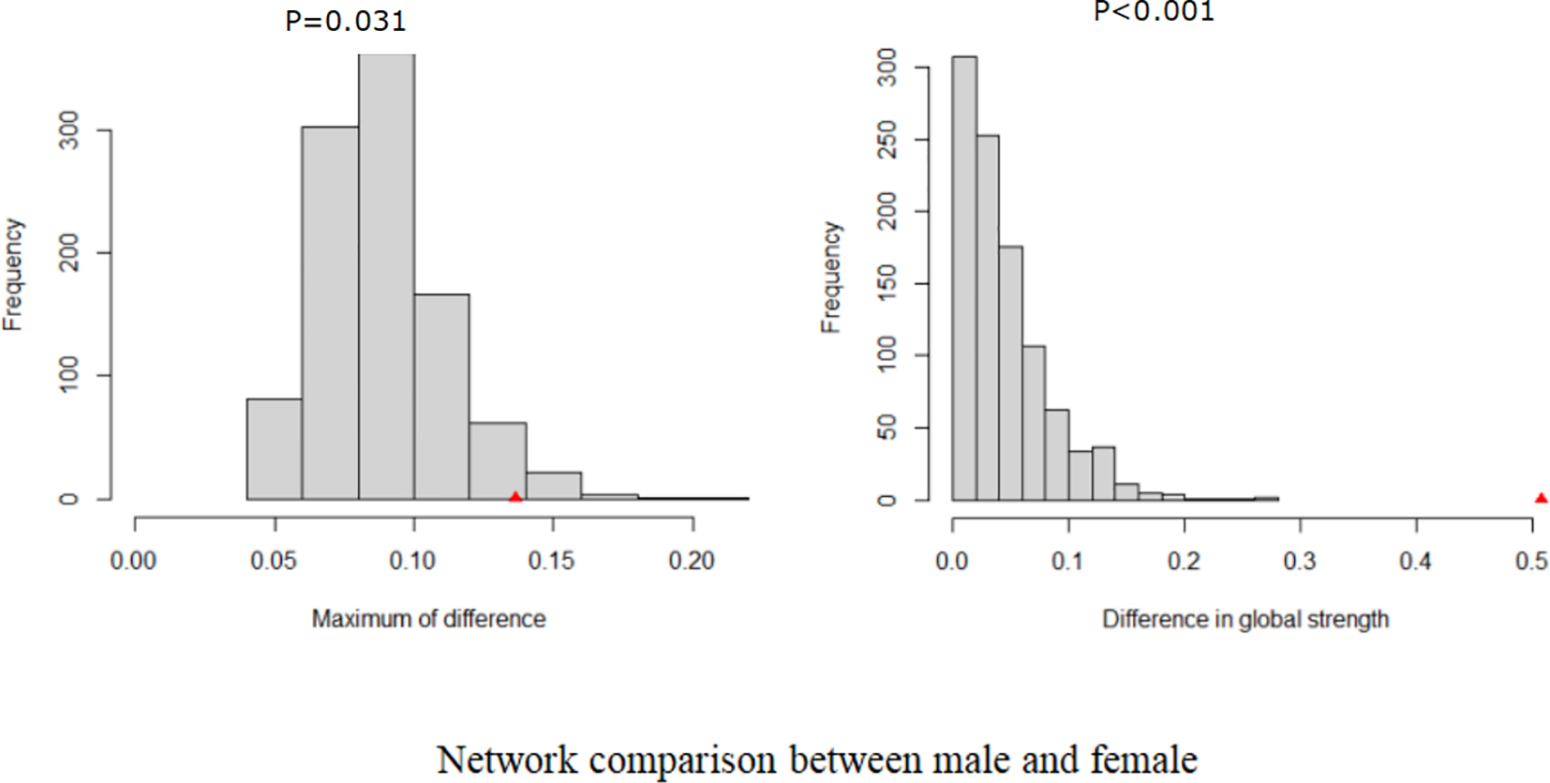
Network comparison.

**Figure 3** presents the comparison of the network models between male and female MHPs. Significant differences were found in network global strength (P < 0.001) and the distribution of edge weights (P = 0.031). Specifically, the female network was sparser than the male network.

### 3.6 Network stability and accuracy

In **Supplementary Figure S1**, the left panel displays the bootstrapped 95% CIs for edge weights in the female network, indicating a reliable network with narrower intervals, thus signifying greater stability. Conversely, the intervals for the male network are wider, suggesting a less stable network than the female network, though still within acceptable limits. As shown in **Supplementary Figure S2**, the CS-coefficients for both female and male EIs were 0.75, indicating that the EI structure of both networks remains unchanged even after removing 75% of the sample, thus confirming their stability. In addition, bootstrapped difference tests for centrality indices revealed that most boxes were grey in the male network, indicating that the confidence interval for the centrality indices in the bootstrap process contains zero, so the value might not be reliable enough. In contrast, most boxes were black in the female network, indicating that the female network was more stable (**Supplementary Figure S3**). Regarding edge weight differences (as shown in **Supplementary Figure S4**), in the male panel, more than half of the edges did not differ significantly from each other.

## 4 Discussion

To the best of our knowledge, this was the first study that compared COVID-19 fear between male and female MHPs with PISC using network analysis. The prevalence of COVID-19 fear in this study was 63.5% (95% CI: 62.3%-64.6%). Due to a lack of studies comparing the prevalence of COVID-19 fear among HPs in China, a direct comparison with similar studies was not possible. Previous research found that MHPs with COVID-19 fear were usually at an increased risk of burnout and secondary traumatic stress (58). Our study adds to this literature by examining the gender differences in COVID-19 fear among MHPs with PISC. The results revealed that female MHPs with PISC (65.0%, 95% CI: 63.7%-66.2%) exhibited a higher prevalence of COVID-19 fear compared to their male counterparts (61.7%, 95% CI: 58.9%-64.4%). The gender discrepancy is partly consistent with previous findings from Saudi Arabia and Belgium that female HPs reported higher levels of COVID-19-related anxiety than their male counterparts (59, 60). Possible reasons for the gender difference might relate to females perceiving the COVID-19 as posing a higher risk to themselves and a greater threat to public health than males (61). Additionally, the hypothalamic-pituitary-adrenal axis in females might be more sensitive, potentially increasing their vulnerability to fear responses (62).

We found that having married status, insomnia, worse economic status, and depression were significantly associated with higher levels of COVID-19 fear in both genders, which aligns with previous studies on factors associated with the perceived COVID-19 fear among Brazilian college students, and the economic and societal influence of the COVID-19 lockdown on the general Japanese population (63, 64). While being married generally enhanced mental health and coping abilities, married MHPs during the pandemic often faced domestic caregiving responsibilities while also managing substantial clinical workloads, thereby exacerbating their perceived vulnerability and pandemic-related fear (65). The association between insomnia and COVID-19 fear among MHPs might be mediated through several factors. Clinically, the need to manage challenging behaviors among patients, including those exhibiting acute psychiatric disturbances, could lead to prolonged occupational stress (66). This heightened stress response could result in increased serum cortisol levels and reduced melatonin synthesis, which might in turn disrupt sleep patterns, initiation, and maintenance, thereby exacerbating insomnia (67). Further, participants with depression were more susceptible to COVID-19 fear, as depressive symptoms might deplete emotional resources, impairing their ability to provide high-quality patient care (68). Additionally, female MHPs with past quarantine experience exhibited higher levels of COVID-19 fear, aligning with findings from dental professionals who reported that quarantine measures were associated with increased pandemic-related fear (69). This heightened fear among female MHPs might be partially attributed to the separate quarantine locations in hotels or hospitals, that were remote from their established social networks. Consequently, they might perceive significantly less social support from colleague and families that is a critical stress-coping mechanism for females, compared to males (70, 71), thus amplifying their fear. Furthermore, quarantine restrictions might limit their ability to share domestic responsibilities with family members and exacerbate their psychological burden. In China, females usually had disproportionately more family and childcare duties (72) and their inability to fulfill these roles during quarantine could likely intensify their distress. Moreover, we found a significant association between lower economic status and heightened fear of COVID-19. A possible explanation might be related to economic vulnerability being a known predictor of poorer mental health outcomes (73). Specifically, MHPs in lower economic status might experience increased anxiety due to uncertainties about the future, which could amplify their fear of COVID-19.

Regarding the network comparison of COVID-19 fear between both genders, the overall structures were similar, but the global strengths differed significantly. Specifically, the female network model exhibited greater sparsity, which might be attributed to gender-specific variations in emotion regulation strategies. For instance, females tended to employ a broader range of emotion regulation strategies, including cognitive reappraisal, problem-solving, acceptance and distraction, compared to males (74). Such enhanced regulatory capacity might result in a more dispersed fear response among females, rather than a concentrated focus on specific fear-related items. Compared to their male counterparts, females tended to maintain broader, non-kin-centered social networks characterized by emotional communication and self-disclosure, which could enhance their access to social support (75). Such support could mitigate the association between specific aspects of fear, thereby weakening the overall connectivity within the female network model (76). Consequently, the global strength of internal connections in the female network model might be reduced.

In the male network, the most central symptom was “Sleep difficulties caused by worried about COVID-19” (FOC6), indicating its pivotal role within the network. This finding is consistent with previous research on COVID-19-related fear among fire service recruits (37). Sleep difficulties, defined as challenges in falling or staying asleep (77), represents a physical manifestation of fear and is associated with somatization symptoms. Such symptoms could significantly impair work performance and overall well-being (78). A nationwide study in China found that approximately 36.2% (95% CI: 35.0%–37.0%) of MHPs experienced insomnia, which was likely exacerbated by heightened work pressure and increased risks of infection during the pandemic (79). Many MHPs had direct care responsibilities for COVID-19 patients despite having limited experience in infectious disease management, which likely intensified their fear and sleep disturbances that had persisted into the post-pandemic era. Male MHPs were disproportionately assigned to leadership positions and high-risk clinical duties during the pandemic, which often led to extended work hours and heightened work pressure (80). Such occupational stressors likely contributed to higher rates of work burnout among male compared to female MHPs, which might have disrupted their sleep patterns (81). Additionally, stigma often prevented males from seeking mental health care (82), which could lead to unaddressed psychological distress manifesting as somatic symptoms.

In the female network model, “Palpitations when thinking about COVID-19” (FOC7) emerged as the most central symptom, which is consistent with a previous network analysis of COVID-19 fear among Brazilian adults (83). Palpitations, defined as the perception of an abnormal heartbeat (84), are strongly associated with intense psychological distress such as fear (85). For instance, a study on female participants found that fear-induced activation of the sympathetic nervous system could elevate heart pump function and induce vasoconstriction, potentially triggering palpitations (86). Additionally, palpitations were among the most common symptoms reported in persons with long COVID (87). In China, the abrupt termination of China’s Zero-COVID Policy and the subsequent surge in COVID-19 cases led to a sharp rise in hospital visits, overwhelming the healthcare systems and disrupting daily life (88). In this context, the ongoing uncertainty and fear of infection had likely aggravated psychological distress, which in turn contributed to persistent symptoms such as palpitations (FOC7). Further, MHPs of whom the majority were female nurses, often faced increased workload and night shifts, as reported in a study on occupational burnout among psychiatric medical staff (89). Night shifts could adversely affect the physical and mental health of MHPs, with palpitations emerging as a predominant concern, thus contributing to this being the most central symptom impacting COVID-19 fear, especially among female MHPs. In addition, research indicated greater emotional self-awareness in females compared with males, including the perception of internal bodily states (e.g., cardiac, respiratory, or gastrointestinal sensations) could be positively correlated with emotional processing capacity (90–92). This increased sensitivity to physical-emotional integration might explain why female MHPs disproportionately reported palpitations during fear states, as they were more attuned to psychosomatic manifestations of distress (93).

The strengths of this study included the large sample size, the unique sample of MHPs and use of the novel statistical approach, network analysis, that could examine the gender-specific COVID-19 fear at a symptom level. Additionally, we explored the correlates across both genders, thereby providing valuable insights into gender differences in fear responses. However, several limitations should be noted. First, as a cross-sectional study, the network structure could not establish either the temporal or causal relationships between symptoms; therefore, longitudinal studies are needed to capture dynamic symptom changes over time. Second, the assessment of COVID-19 fear was based on self-report, which might introduce recall bias. Third, the data collected on quarantine exposure did not distinguish between different quarantine settings (e.g., home vs. centralized facilities), which might influence mental health outcomes. Future studies should capture such details to understand context-dependent effects. Third, the use of an online survey might result in selection bias, thus limiting the generalizability of the findings. Fourth, due to the exploratory nature of the study (94, 95), the significance level was unadjusted for multiple comparisons, although this might potentially increase the risk of false positive results.

In conclusion, this network analysis found that COVID-19 fear was more common among female MHPs compared with male MHPs. Having married status, insomnia, worse economic status and depression were significantly associated with higher levels of COVID-19 fear in both genders. According to the network analysis, “Sleep difficulties caused by worried about COVID-19” (FOC6) was the most central symptom in the male network model while “Palpitation when thinking about COVID-19” (FOC7) was the most central symptom in the female model. Relevant interventions targeting such symptoms should be implemented in the post-pandemic era to alleviate COVID-19 fear effectively and improve the mental health of MHPs.

## Data Availability

The datasets presented in this article are not readily available because the Research Ethics Committee of Beijing Anding Hospital that approved the study prohibits the authors from making publicly available the research dataset of clinical studies. Requests to access the dataset should be addressed to the corresponding author.
