# Correction: Gender differences in the prevalence and correlates of COVID-19 fear among mental health professionals: a network perspective based on a national survey in China

**DOI:** 10.3389/fpsyt.2025.1716267

**Published:** 2025-10-09

**Authors:** Shu-Ying Rao, Mu-Rui Zheng, Feng-Rong An, Yuan Feng, Zhaohui Su, Teris Cheung, Gabor S. Ungvari, Chee H. Ng, Yu-Tao Xiang, Gang Wang

**Affiliations:** ^1^ Unit of Psychiatry, Department of Public Health and Medicinal Administration, & Institute of Translational Medicine, Faculty of Health Sciences, University of Macau, Macao, Macao SAR, China; ^2^ Centre for Cognitive and Brain Sciences, University of Macau, Macao, Macao SAR, China; ^3^ Beijing Key Laboratory of Mental Disorders, National Clinical Research Center for Mental Disorders & National Center for Mental Disorders, Beijing Anding Hospital, Capital Medical University, Beijing, China; ^4^ School of Public Health, Southeast University, Nanjing, China; ^5^ School of Nursing, Hong Kong Polytechnic University, Hong Kong, Hong Kong SAR, China; ^6^ Section of Psychiatry, University of Notre Dame Australia, Fremantle, WA, Australia; ^7^ Division of Psychiatry, School of Medicine, University of Western Australia, Perth, WA, Australia; ^8^ Department of Psychiatry, The Melbourne Clinic and St Vincent’s Hospital, University of Melbourne, Richmond, VIC, Australia

**Keywords:** mental health professionals, gender difference, fear of COVID-19, network analysis, mental health

There was an error in the Affiliations. Author “Gang Wang” was erroneously assigned to affiliation 4, “School of Public Health, Southeast University, Nanjing, China”. This should be affiliation 3, “Beijing Key Laboratory of Mental Disorders, National Clinical Research Center for Mental Disorders & National Center for Mental Disorders, Beijing Anding Hospital, Capital Medical University, Beijing, China.”

The original version of this article has been updated.

