# Supplementary material for: Gender differences in the prevalence and correlates of COVID-19 fear among mental health professionals: a network perspective based on a national survey in China
Source: Front Psychiatry. 2025 Aug 21;16:1631050. doi: 10.3389/fpsyt.2025.1631050 (PMC12408580; doi:10.3389/fpsyt.2025.1631050)
Supplement: Supplementary file 1 [file SupplementaryFile1.docx]

**Supplementary Material**

Table S1. Descriptive information and network centrality indices of mental health professionals by gender

Table S2. Network comparison of edge difference

Figure S1. Edge-weight confidence interval of the fear of COVID-19 network model in mental health professionals by gender

Figure S2. Network stability of the fear of COVID-19 network model in mental health professionals by gender

Figure S3. Bootstrapped difference test for EI by gender

Figure S4. Estimation of edge weight difference by bootstrapped difference test by gender

Table S1. Descriptive information and network centrality indices of mental health professionals by gender

| Item | Item content | Female | | | Male | | | | Difference test test |  | |
| --- | --- | --- | --- | --- | --- | --- | --- | --- | --- | --- | --- |
|  |  | Mean (SD) | Predictability | EI | | Mean (SD) | EI | Predictability | C | | P |
| FOC1 | Afraid of COVID-19 | 3.3(0.7) | 0.8 | 0.60 | | 2.6(1.1) | 0.75 | 0.6 | -0.15 | | <0.001 |
| FOC2 | Uncomfortable to think about COVID-19 | 3.3(0.7) | 0.8 | 0.70 | | 2.7(1.1) | 0.84 | 0.5 | -0.14 | | <0.001 |
| FOC3 | Clammy when thinking about COVID-19 | 2.8(0.7) | 0.8 | 0.75 | | 2.3(1.0) | 1.02 | 0.4 | -0.27 | | 1.000 |
| FOC4 | Afraid of losing life because of COVID-19 | 2.9(0.8) | 0.8 | 0.55 | | 2.3(1.0) | 0.73 | 0.5 | -0.18 | | <0.001 |
| FOC5 | Nervous when watching news about COVID-19 | 3.1(0.7) | 0.7 | 0.81 | | 2.5(1.0) | 1.01 | 0.4 | -0.2 | | 1.000 |
| FOC6 | Sleep difficulties caused by worried about COVID-19 | 2.9(0.7) | 0.7 | 0.87 | | 2.3(1.0) | 1.08 | 0.4 | -0.21 | | <0.001 |
| FOC7 | Palpitation when thinking about COVID-19 | 2.9(0.8) | 0.6 | 1.03 | | 2.3(1.0) | 1.03 | 0.4 | 0.00 | | 1.000 |
| Note: SD: standard deviation; EI: Expected influence | | | | | | | | |  |  | |

Table S2. Network comparison of edge difference.

| Edge difference p-value | | |
| --- | --- | --- |
| Node1 | Node2 | Male to female fefemalefemale female female |
| FOC1 | FOC2 | 0.310 |
| FOC1 | FOC3 | 0.805 |
| FOC2 | FOC3 | 0.147 |
| FOC1 | FOC4 | 0.462 |
| FOC2 | FOC4 | 0.210 |
| FOC3 | FOC4 | **0.003** |
| FOC1 | FOC5 | 0.423 |
| FOC2 | FOC5 | 0.940 |
| FOC3 | FOC5 | 0.411 |
| FOC4 | FOC5 | 0.328 |
| FOC1 | FOC6 | 0.409 |
| FOC2 | FOC6 | 0.869 |
| FOC3 | FOC6 | **0.013** |
| FOC4 | FOC6 | 0.553 |
| FOC5 | FOC6 | 0.164 |
| FOC1 | FOC7 | 0.518 |
| FOC2 | FOC7 | 0.291 |
| FOC3 | FOC7 | **0.027** |
| FOC4 | FOC7 | 0.861 |
| FOC5 | FOC7 | **0.021** |
| FOC6 | FOC7 | 0.383 |

Figure S1. Edge-weight confidence interval of the fear of COVID-19 network
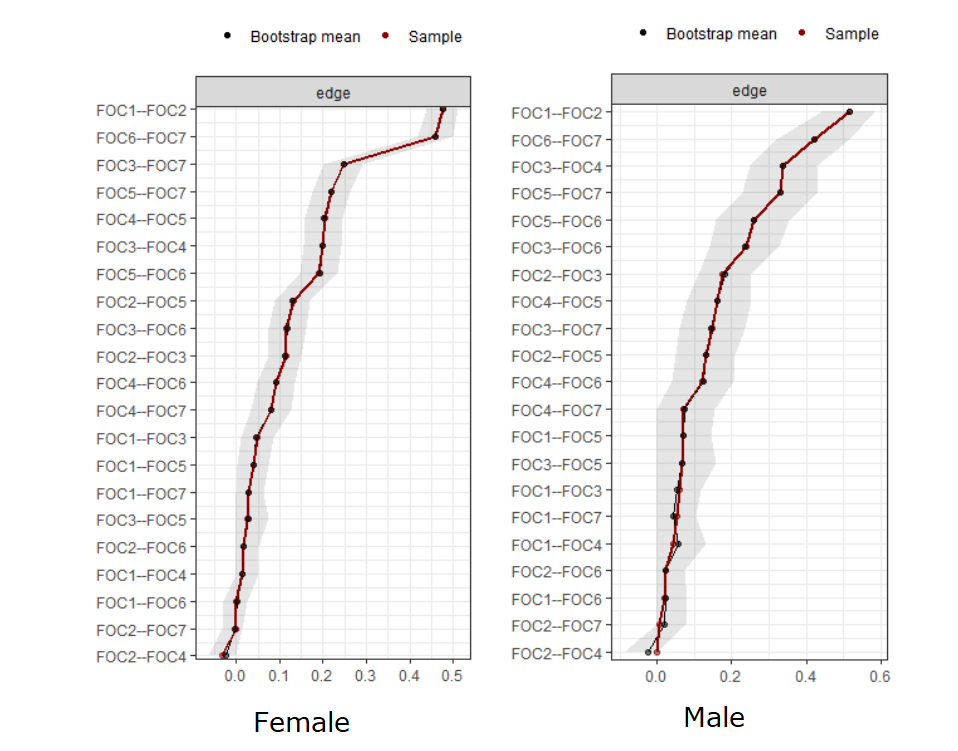
model in mental health professionals by gender

Notes: The black dots indicate the values of each edge weight, ordered from the highest to the lowest value. The gray area represents the 95% confidence intervals of edge weights, estimated with the non-parametric bootstrap procedure. (FOC1: Afraid of COVID-19; FOC2: Uncomfortable to think about COVID-19; FOC3: Clammy when thinking about COVID-19; FOC4: Afraid of losing life because of COVID-19; FOC5: Nervous when watching news about COVID-19; FOC6: Sleep difficulties caused by worried about COVID-19; FOC7: Palpitation when thinking about COVID-19)

Figure S2 Network stability of the fear of COVID-19 network model in mental health professionals by gender


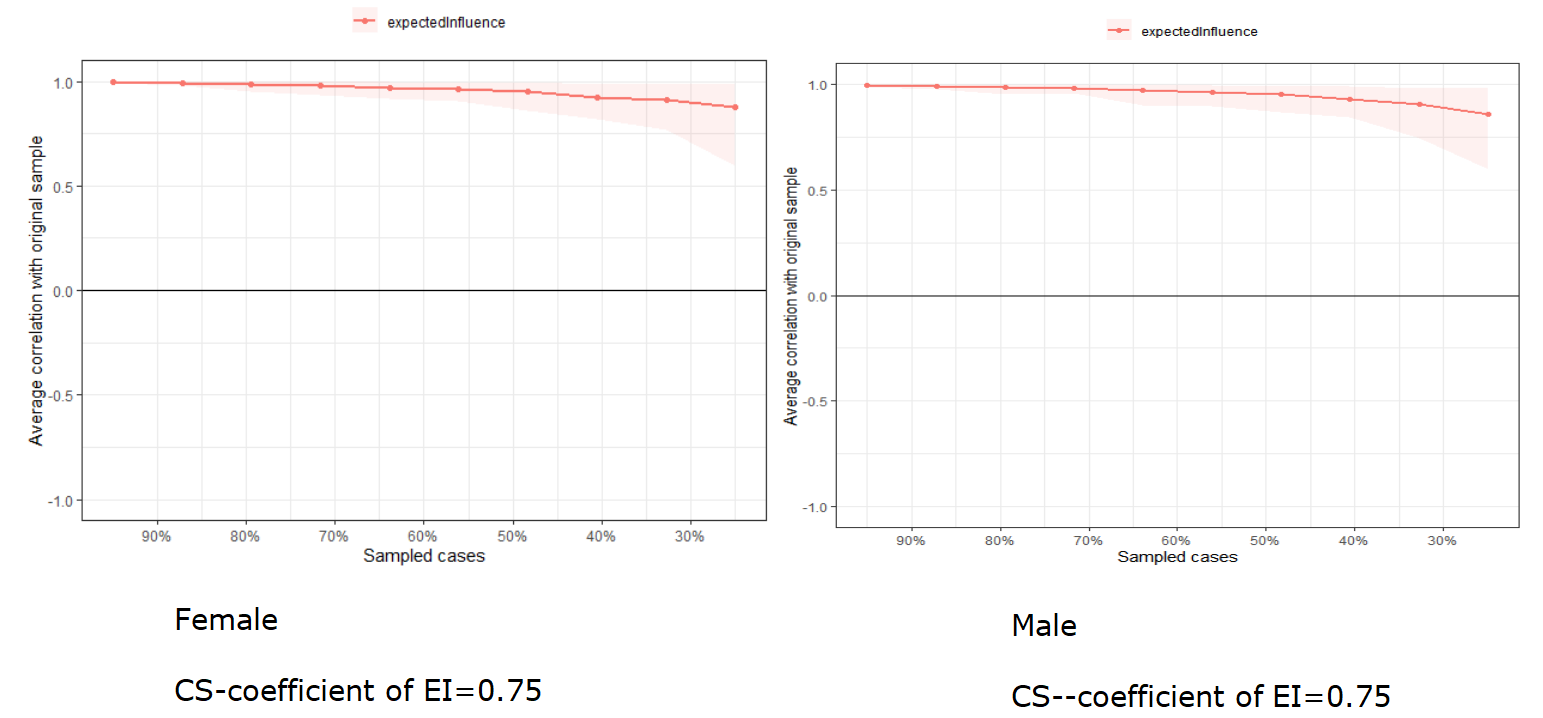


Female CS-coefficient of EI=0.75

Male CS-coefficient of EI=0.75

Figure S3. Bootstrapped difference test for centrality indices by gender


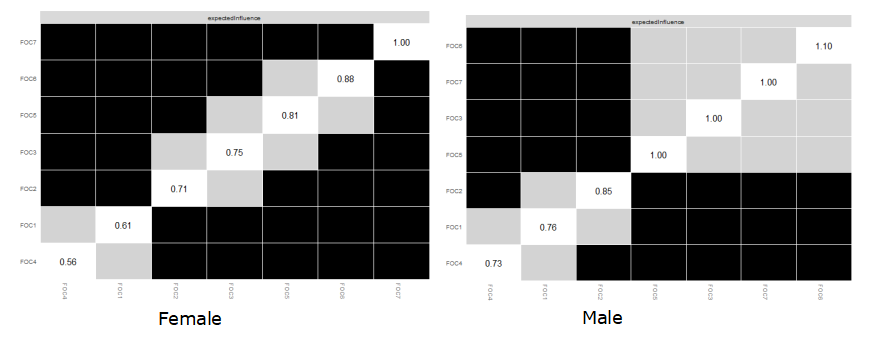


Notes: Gray boxes indicate that centrality indices do not significantly differ between the pair of nodes. Black boxes represent centrality indices with significant difference between the pair of nodes (α = 0.05).

Figure S4. Estimation of edge weight difference by bootstrapped difference test by gender


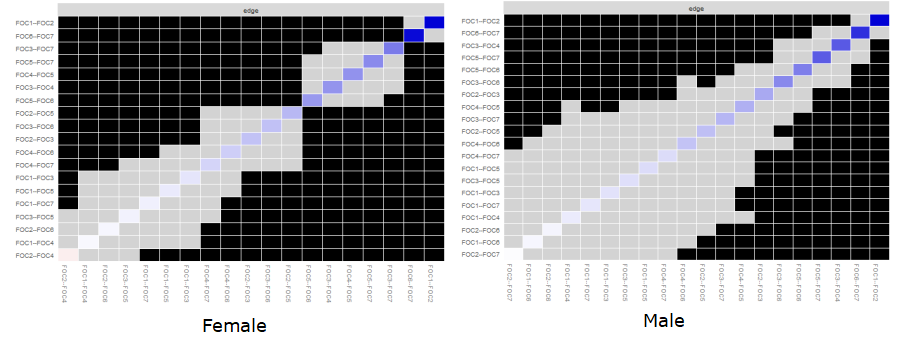


Notes: Gray boxes indicate edges that do not significantly differ from one-another. Black boxes represent edges with significant difference from one another (α=0.05). Blue boxes in the edge-weight plot indicate positive correlations.
